# Multi-locus phylogeny using topotype specimens sheds light on the systematics of *Niviventer* (Rodentia, Muridae) in China

**DOI:** 10.1186/s12862-016-0832-8

**Published:** 2016-12-01

**Authors:** Bin Zhang, Kai He, Tao Wan, Peng Chen, Guozheng Sun, Shaoying Liu, Truong Son Nguyen, Liangkong Lin, Xuelong Jiang

**Affiliations:** 1 State Key Laboratory of Genetic Resources and Evolution, Kunming Institute of Zoology, Chinese Academy of Sciences, Kunming, Yunnan China; 2 Kunming College of Life Science, University of Chinese Academy of Sciences, Kunming, Yunnan China; 3 National Museum of Natural History, Smithsonian Institution, Washington D.C., USA; 4 Chengdu Research Base of Giant Panda Breeding, Sichuan Key Laboratory of Conservation Biology for Endangered Wildlife, Chengdu, Sichuan China; 5 China Forest Exploration and Design Institute on Kunming, Kunming, Yunnan China; 6 Sichuan Academy of Forestry, Chengdu, Sichuan China; 7 Department of Vertebrate Zoology Institute of Ecology and Biological Resources, Vietnam Academy of Sciences and Technology, Hanoi, Vietnam; 8 Department of Life Sciences, Laboratory of Wildlife Ecology, Tunghai University, Taichung, Taiwan; 9 The Kyoto University Museum, Kyoto University, Kyoto, 606-8501 Japan

**Keywords:** Cryptic species, Species delimitation, *Niviventer*, Phylogenetics, Taxonomy, Topotype

## Abstract

**Background:**

*Niviventer* is a genus of white-bellied rats that are among the most common rodents in the Indo-Sundaic region. The taxonomy of the genus has undergone extensive revisions and remains controversial. The current phylogeny is unresolved and was developed primarily on the basis of mitochondrial genes. Identification is extremely difficult, and a large number of GenBank sequences seem to be problematic. We extensively sampled specimens of *Niviventer* in China and neighboring northern Vietnam, including topotypes of the most reported species (*n* = 6), subspecies (*n* = 8), and synonyms (*n* = 4). We estimated phylogenetic relationships on the basis of one mitochondrial and three nuclear genes, using concatenation and coalescent-based approaches. We also employed molecular species delimitation approaches to test the existence of cryptic and putative new species.

**Results:**

Our phylogeny was finely resolved, especially for the *N. confucianus*-like species. Our data provided the first support for *N. brahma* and *N. eha* as sister species, an assignment that is congruent with their morphological similarities. Species delimitation analyses provided new insight into species diversity and systematics. Three geographic populations of *N. confucianus* and one of *N. fulvescens* were supported as genetically distinct in our species delimitation analyses, while three recognized species (*N. coninga*, *N. huang*, and *N. lotipes*) were not strongly supported as distinct.

**Conclusions:**

Our results suggested that several genetically distinct species may be contained within the species currently known as *N. confucianus* and *N. fulvescens*. In addition, the results of Bayesian Phylogenetics and Phylogeography (BPP) for *N. coninga*, *N. huang*, and *N. lotipes* indicated that either inter-specific gene flow had occurred or imperfect taxonomy was present. Morphological examinations and morphometric analyses are warranted to examine the molecular results.

**Electronic supplementary material:**

The online version of this article (doi:10.1186/s12862-016-0832-8) contains supplementary material, which is available to authorized users.

## Background

Taxonomy and systematics are crucial for understanding biological diversity and the conservation of species. Nonetheless, a complete understanding of species diversity remains a distant goal for most groups of organisms. A recent review of newly discovered mammal species showed that saturation of species discovery in Mammalia has not been reached. The newly added species include not only new described species, but also revisionary new species, which are synonyms or subspecies that have been recognized as valid species [1]. The latest version of *Mammal Species of the World* (2005) recognized 5,339 valid species, as well as 6,351 subspecies and 15,881 species-level synonyms [2]. A large proportion of synonyms and subspecies are assigned those ranks because of the lack of specimens, comprehensive analyses, and critical evaluation. This is especially the case for taxa that are widely distributed and morphologically unremarkable, such as the white-bellied rats of the genus *Niviventer*. These murine rodents are small to medium-sized and are mainly distributed in China, southern Himalaya, and Southeast Asia in various habitats over a wide range of elevations [3, 4]. While most species of *Niviventer* have very limited distributions, a few species, such as *N. cremoriventer, N. confucianus*, and *N. fulvescens,* are widely spread across large geographic areas and various landscapes [5–7].

The taxonomy of *Niviventer* has a long and complicated history. Representatives of *Niviventer* were originally included in the genus *Mus* (1836–1911), after which they were placed in *Epimys* (1911–1916), and *Rattus* (1916–1981; *Rattus* = *Epimys*, Hollister [8]), successively. The genus was established by Musser [3], who included 15 species and divided the genus into the *N. andersoni*-division and *N. niviventer*-division. According to the taxonomy presented by Musser and Carleton in 2005 [9], 17 taxa had full species status, while another 65 were recognized as subspecies or synonyms. The widespread *N. cremoriventer*, *N. confucianus*, and *N. fulvescens* had 10, 14, and 26 subspecies and synonyms, respectively. Recent studies integrating karyotypic information, DNA sequences, and morphological characters recognized *N. lotipes* (a synonym of *N. tenaster*) [10], *N. huang*, and *N. bukit* (synonyms of *N. fulvescens*) as full species [11] but placed *N. langbianis* into *Chiromyscus* [12]. Molecular phylogenetic studies further found that the widely distributed species were paraphyletic or even polyphyletic (e.g., *N. andersoni, N. confucianus, N. excelsior,* and *N. fulvescens*) [13]. Despite the possibility of misidentification, the number of putative species always exceeded that of recognized species [13, 14], implying that species diversity was underestimated. Thus, it was reasonable to re-evaluate the large body of remaining subspecies/synonyms within this genus.

The complex distribution pattern, large number of subspecies/synonyms, and morphological similarities make taxonomic and systematic revision for the genus *Niviventer* extremely difficult. Many species are morphologically very similar to one another [3]. For example, geometric morphometric analyses in a previous study showed that two distinctive genetic lineages largely overlapped (i.e., *N. confucianus* and *N. fulvescens*; Fig. 6 in Lu et al. [14]). Examination of type specimens is always crucial for taxonomic revision and essential for re-evaluating the synonyms. However, the type series for most species distributed in Asia are deposited in natural history museums in Europe and the United States. Thus, comprehensive genetic analyses using topotypes may uncover cryptic species diversity and help to determine key taxa that need to be diagnosed before the type series is examined. In addition to the existing taxonomic problems, the phylogeny of *Niviventer* remained largely obscure. Although several major clades and species groups have been recognized, the relationships among them were unresolved, and the phylogenetic positions of *N. coninga* and *N. culturatus* were contradictory [11, 15]. More than 540 *Niviventer* specimens have been sequenced, but most of the previous studies were conducted using only mitochondrial cytochrome b (*CYT B*) and cytochrome c oxidase (*COI*) genes [11, 13, 15, 16]. In another previous study using both mitochondrial (*CYT B, COI*, and *D-loop*) and nuclear (*IRBP*) loci, the gene trees were conflicting, suggesting that inclusion of multiple nuclear genes is warranted [14].

According to the taxonomy of Musser and Carleton [9] and recent studies [10–12], there are 10 recognized *Niviventer* species distributed in China, accounting for 55% of *Niviventer* species, including 5 endemic to China (*N. andersoni*, *N. coninga*, *N. culturatus*, *N. excelsior,* and *N. lotipes*) and 5 both inside and outside China (*N. brahma*, *N. eha*, *N. confucianus*, *N. fulvescens*, and *N. huang*). Nine *Niviventer* species (*N. cremoriventer*, *N. cameroni*, *N. rapit*, *N. fraternus*, *N. lepturus*, *N. hinpoon*, *N. tenaster*, *N. bukit,* and *N. niviventer*) are distributed in Southeast Asia outside China. Another 24 taxa are recognized as synonyms or subspecies of *N. andersoni* (*n* = 3), *N. confucianus* (*n* = 14), *N. eha* (*n* = 1), *N. excelsior* (*n* = 1), *N. fulvescens* (*n* = 1), and *N. huang* (*n* = 4). Moreover, according to Lu et al. [14], undescribed species may exist in the mountains of southwestern China, an area characterized by extremely complex topography [17].

In the present study, we extensively sampled white-bellied rats throughout China and neighboring northern Vietnam, capturing topotypes of 18 species, subspecies, and synonyms. We sequenced four unlinked loci, reconstructed phylogenetic relationships, and delimited species boundaries. The goals were i) to clarify the status of species, subspecies, and synonyms, and ii) to uncover underestimated species diversity.

## Results

### Genetic sequences

**Fig. 1:**
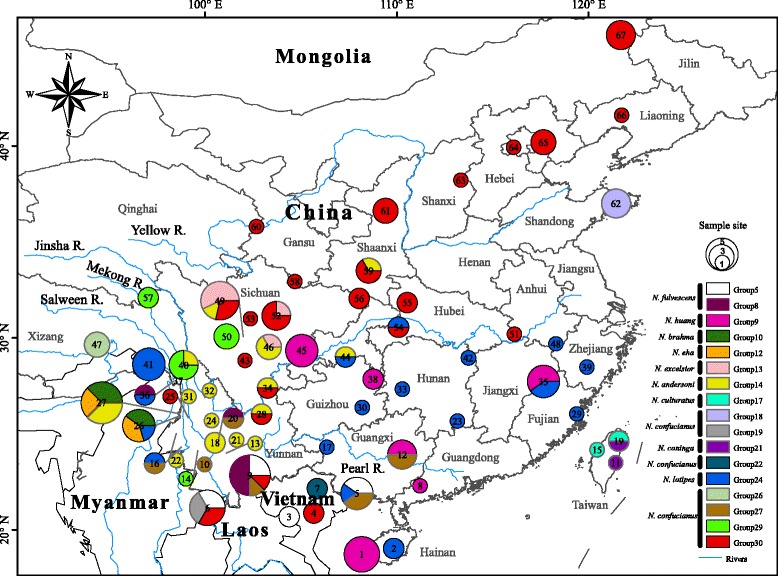
Sample localities of specimens used in the present study. The numbers correspond to the locality numbers in Additional file 1: Table S1

A total of 157 *Niviventer* individuals representing 10 species were collected from China and neighboring northern Vietnam (Fig. 1, Table 1, Additional file 1: Table S1). We obtained 4,278 bp of sequences from 147 specimens, including 1,140 bp of mitochondrial DNA (mtDNA; *CYT B* [1,140 bp]) and 3,138 bp of nuclear genes (*GHR* [768 bp], *IRBP* [1,149 bp], and *RAG1* [1,221 bp]). The other ten individuals each failed to amplify one of the three nuclear genes. The new sequences have been deposited in GenBank under the accession numbers KY068361 to KY069019 (Additional file 1: Table S1). The appearance of premature stop codons or frame-shift mutations was not observed in coding regions. In addition, 537 sequences (517 *CYT B*, 8 *GHR*, and 12 *IRBP*) representing 517 individuals of 17 *Niviventer* spp. (including *N. andersoni*, *N. brahma*, *N. confucianus*, *N. coninga*, *N. culturatus*, *N. eha*, *N. excelsior*, *N. fulvescens*, *N. huang*, *N. bukit*, *N. hinpoon*, *N. niviventer*, *N. cremoriventer*, *N. tenaster*, *N. rapit*, *Niviventer* sp. 1 and *Niviventer* sp. 2 [18]) were downloaded from GenBank (Additional file 2: Table S2). Sequences of *Leopoldamys edwardsi*, *L. neilli*, *Rattus andamanensis*, *R. norvegicus*, *Chiromyscus chiropus*, *C. thomasi*, *C. langbianis*, and *Mus musculus* were chosen as outgroups (Additional file 2: Table S2).

**Table 1:** The current taxonomy of the *Niviventer* genus distributed in China. All subspecies and synonyms are included. Taxa and topotypes included in the present study are shown. Sample locality numbers refer to those in Fig. 1 and Additional file 1: Table S1

Status	Taxon	Type Locality	Included	Topotypes (sample locality number)
Species	*N. andersoni* (Thomas, 1911)	Mt. Emei, Sichuan, China	Y	Y (46)
Subspecies	*N. andersoni pianmaensis* Li & Yang, 2009	Pianma, Yunnan, China	Y	Y (27)
Subspecies	*N. andersoni ailaoshansis* Li & Yang, 2009	Mt. Ailao, Yunnan, China	Y	Y (13, 18, 21)
Synonym	*N. andersoni lushuiensis* Wu & Wang, 2002	No specified	N	N
Species	*N. brahma* (Thomas, 1914)	Mishmi Hills, Arunachal Pradesh	Y	N
Species	*N. confucianus* (Milne-Edwards, 1871)	Baoxing, Sichuan, China	Y	Y (52)
Synonym	*Rattus confucianus littoreus* Cabrera, 1922	Fuzhou, Fujian, China	N	N
Synonym	*Epimys zappeyi* Allen, 1912	Mt. Wa, Sichuan, China	N	N
Synonym	*Rattus confucianus yaoshanensis* Shih, 1930	Luoxiang, Guangxi, China	N	N
Synonym	*Rattus confucianus sinianus* Shih, 1931	Lechang, Guangdong, China	N	N
Synonym	*Rattus elegans* Shih, 1931	Lechang, Guangdong, China	N	N
Subspecies	*N. confucianus sacer* (Thomas, 1908)	Yantai, Shandong, China	Y	Y (62)
Synonym	*Mus confucianus luticolor Thomas,*1908	Yan’an, Shaanxi, China	Y	Y (61)
Synonym	*Epimys confucianus canorus* Thomas, 1922	Wenxian, Gansu, China	Y	Y (58)
subspecies	*N. confucianus chihliensis* (Thomas, 1917)	Eastern Tombs, Hebei, China	Y	Y (65)
subspecies	*N. confucianus mentosus* (Thomas, 1916)	Hkampti, Myanmar	Y	N
subspecies	*N. confucianus yushuensis* (Wang & Zheng, 1981)	Yushu, Qinghai, China	Y	Y (57)
subspecies	*N. confucianus naoniuensis* (Zhang & Zhao, 1984)	Mt. Naoniu, Baicheng, Jilin, China	Y	Y (67)
subspecies	*N. confucianus yajiangensis,* Deng & Wang, 2000	Bajiaolou, Yajiang, Sichuan, China	Y	Y (50)
subspecies	*N. confucianus deqinensis,* Deng & Wang, 2000	Adong, Deqin, Yunnan, China	Y	Y (40)
Species	*N. coninga* (Swinhoe, 1864)	Taiwan, China	Y	Y (11, 19)
Species	*N. culturatus* (Thomas, 1917)	Taiwan, China	Y	Y (15, 19)
Species	*N. eha* (Wroughton, 1916)	Lachen, Sikkim, India	N	N
Subspecies	*N. eha ninus* (Thomas, 1922)	Salween-Mekong divide, Yunnan, China	Y	N
Species	*N. excelsior* (Thomas, 1911)	Kangding, Sichuan, China	Y	Y (49)
Synonym	*N. excelsior tengchongensis,* Deng & Wang, 2002	No specified	N	N
Species	*N. fulvescens* (Gray, 1847)	Katmandu, Nepal	Y	N
Synonym	*Rattus huang vulpicolor* Allen, 1926	Nating River, Yunnan, China	N	N
Species	*N. huang* (Bonhote, 1905)	Guadun, Fujian, China	Y	Y (35)
Synonym	*Mus ling* Bonhote, 1905	Chungfengling, Fujian, China	Y	Y (^a^)
Synonym	*Rattus flavipilis* Shih, 1930	Luoxiang, Guangxi, China	Y	Y (12)
Synonym	*Rattus flavipilis minor* Shih, 1930	Luoxiang, Guangxi, China	Y	Y (12)
Synonym	*Rattus wongi* Shih, 1931	Lechang, Guangdong, China	N	N
Species	*N. lotipes* (Allen, 1926)	Nada, Hainan, China	Y	N

^a^: The topotype of *N. ling* was sequenced in Lu et al. (2015) [14] and was included in the present study. Sample locality is not shown in Fig. 5

### Mitochondrial tree and species recovery

**Fig. 2:**
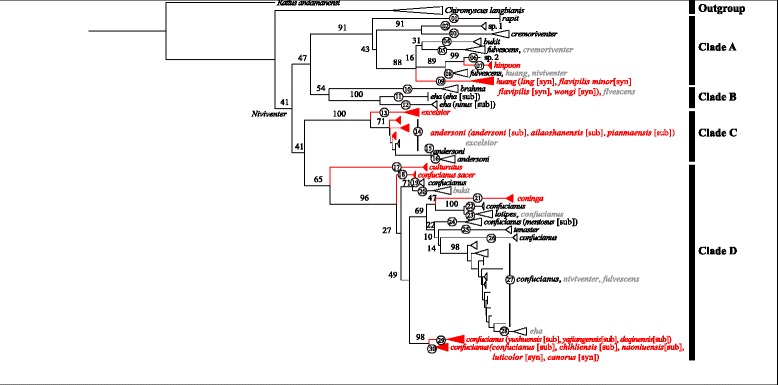
Result of RAxML phylogenetic analyses of the *CYT B* gene. Node numbers indicate bootstrap values. Branch numbers represent putative species recognized by ABGD analyses. Clades in red color indicate topotypes included. Names after each clade are species names (subspecies names [sub] and synonym names [syn]) identified in this study and previous studies (sequences downloaded from NCBI GenBank). Names in black indicate irrefutable identification and in gray indicate likely misidentifications

The best-fit partitioning schemes and substitution models were determined by Partitionfinder V1.0.0 [19] under Bayesian Information Criterion (BIC) (Additional file 3: Table S3). The maximum likelihood (ML) tree calculated from *CYT B* sequences shows the 674 *Niviventer* individuals (Table 2) clustered into four major clades (i.e., clades A–D; Fig. 2), among which relationships were only weakly supported (bootstrap value, BS ≤ 47). Clade A was composed of *N. fulvescens*-like species (including *N. bukit* and *N. huang*) and three Southeast Asian species (*N. rapit*, *N. hinpoon*, and *N. cremoriventer*). Clade B was composed of two species from southwestern China and South Asia (*N. eha* and *N. brahma*). Clade C included *N. andersoni* and *N. excelsior*, representing the *N. andersoni*-division. All individuals in clade D were *N. confucianus*-like species, including subspecies and synonyms of *N. confucianus*, species previously included in *N. confucianus* (i.e., *N. coninga*, *N. culturatus*, and *N. lotipes*), and *N. tenaster*, which was morphologically similar to *N. confucianus*. In addition, individuals identified as *N. bukit*, *N. eha*, *N. fulvescens*, and *N. niviventer* in GenBank were recovered within two major clades each. The Kimura-2-parameter (K2P) distances calculated in MEGA version 7 [20] between candidate species were 0.024 to 0.188 (Additional file 4: Table S4).

**Table 2:** Sample sizes and species used for each analysis

Data set	Sample size (from China)	Species sampling (from China)	CYTB	GHR	IRBP	RAG1	Analyses	Data source(s) (current study + GenBank)
Collected for this study	157 (153)	10 (10)	157	154	154	152	all	/
Download from GenBank	517 (409)	17 (10)	517	8	12	/	ML and ABGD	/
*CYT B* data set	674 (562)	18(10)	674	/	/	/	ML and ABGD	157 + 517
Concatenated data set	204 (176)	18 (10)	204	162	166	152	ML and MrBayes	157 + 47
Full data set	147 (143)	18 (10)	147	147	147	147	BEAST, *BEAST, and BPP	147 + 0

The species delimitation approach Automatic Barcode Gap Discover (ABGD) can partition sequences into groups or putative species based on gaps between intra- and interspecific diversity of barcoded genes [21]. In this study, five ABGD analyses with different relative gap width values (X = 0.5, 1, 1.5, 2, and 2.5) were performed on 674 *CYT B* sequences, and two gaps (*distance* = 0.056 and 0.121) were observed. Nevertheless, all analyses consistently supported a 30-group (i.e., candidate species) scenario only when *intraspecific divergence (p)* = 0.0121 (Table 3; Fig. 2). In this scenario, 589 Chinese sequences were included in 22 groups (group 5, 8–19, 21–24, and 26–30; Additional file 1: Table S1 and Additional file 2: Table S2), and 157 sequences in the current study were included in 18 groups (excluding group 11 labeled *N. eha eha*, and including XZ-YD49 and XZ-YD50 from [14]; group 15 labeled *N. andersoni*, 04047 from [15]; group 16 labeled *N. andersoni,* 002 and 003 from [15]; and group 28 labeled *N. eha*, GLGS103 and GLGS104 from [15]; Additional file 1: Table S1). Two candidate species (groups 14 and 27) did not appear as monophyletic groups in our *CYT B* gene tree. Specimens identified as *N. andersoni*, *N. bukit*, *N. confucianus*, *N. cremoriventer*, *N. eha*, *N. excelsior*, *N. fulvescens*, *N. huang*, and *N. niviventer* in GenBank were each found in at least two groups.

**Table 3:** Results of Automatic Barcode Gap Discovery (ABGD) analyses with K80 distance model

X (Gap width)	*p* = 0.0056	*p* = 0.0121
0.5	53	30
1.0	48	30
1.5	41	30
2.0	41	30
2.5	36	30

The number of putative species are calculated based on different widths of gaps (X), and priors of intraspecific divergence (p)

### Multilocus gene trees

**Fig. 3:**
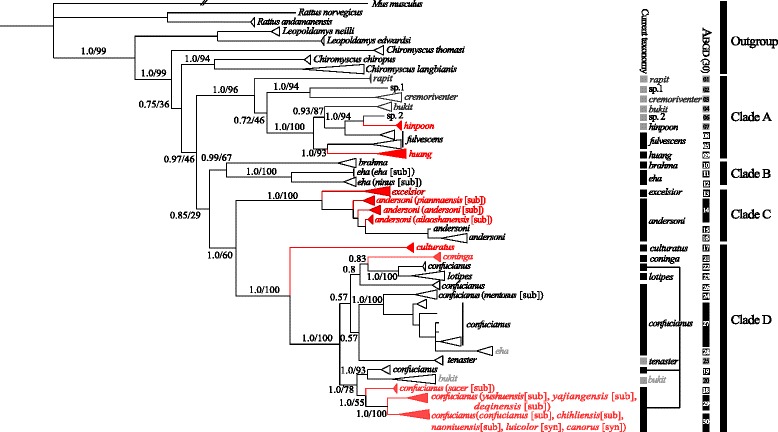
Multilocus phylogeny of *Niviventer* conducted using MrBayes. Node numbers indicate posterior probabilities with bootstrap support. Clades in red indicate topotypes included. The current taxonomy (sequences from GenBank following the identification of previous studies) and putative species recognized by ABGD are presented

The phylogeny constructed from Bayesian and ML analyses of the mitochondrial-nuclear concatenated data (Table 2) resulted in very similar topologies, and only the MrBayes tree is shown (Fig. 3). *Chiromyscus langbianis* and *C. chiropus* were supported as sister species (posterior probabilities [PP] = 1.0, BS = 94), forming the sister clade to *Niviventer* (PP = 0.75, BS = 36; Fig. 3). The four mitochondrial clades of *Niviventer* were also recovered. Compared to the *CYT B* gene tree, interspecific relationships were overall highly supported in clades A, B, and C, but remained unresolved in clade D where only posterior probabilities are noted.

**Fig. 4:**
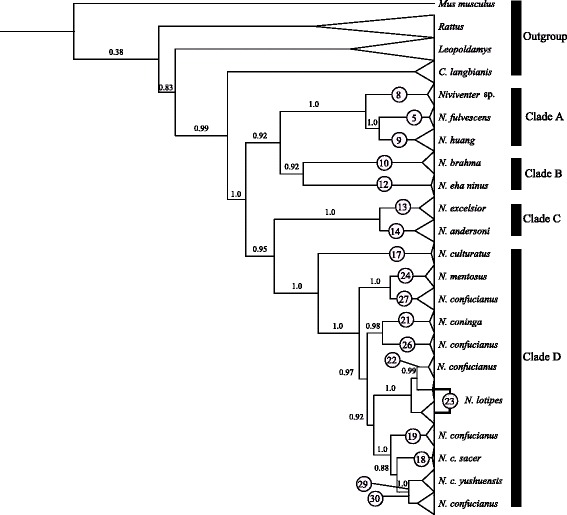
Multilocus phylogenetic tree of full data set using BEAST. Numbers above the nodes indicate Bayesian posterior probabilities (BPP)

The phylogeny using 147 *Niviventer* sequences plus the outgroup (Fig. 4) was constructed according to the mtDNA and concatenated data tree with better support for the relationships within the genus. In particular, the relationships among all clades and within clade D were better resolved in this phylogeny (Fig. 4).

### Species trees and species delimitation

**Fig. 5:**
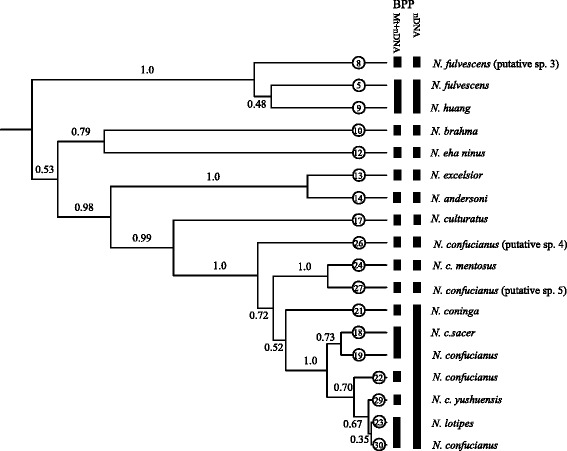
A coalescent-based species tree referred using the *BEAST model based on the result of ABGD analyses. The putative species recognized by Bayesian Phylogenetics and Phylogeography (BPP) using mitochondrial and nuclear genes and using nuclear genes alone are presented. Node numbers indicate posterior probabilities estimated in *BEAST

The species tree simulated in *BEAST showed similar topologies with the BEAST trees (Fig. 5), which assigned 147 individuals from the full data set (Table 2) into 18 groups according to the ABGD results. However, the sister-relationship of groups 5 and 9 and the interspecific relationships in clade D were not robustly supported (PP = 0.35–0.73). Furthermore, group 26 was strongly supported as a sister group to the other *N. confucianus* groups, excluding *N. culturatus* (PP = 1). A sister relationship between groups 24 and 27 was strongly supported (PP = 1), although their position was not stable (PP = 0.72).

The BPP analyses based on the full data set provided similar results when different algorithms, priors, and starting trees were used, and high effective sample sizes (ESSs) (≥200) were observed for all parameters. We adopted a conservative criterion, considering PP ≥ 0.95 as strong support for a putative species (Table 4; Additional file 5: Table S5). When both mitochondrial and nuclear genes were used, 15 putative species were recognized (Fig. 5; Table 4; Additional file 5: Table S5); the nuclear genes alone supported an 11-species scenario (Fig. 5; Table 4; Additional file 5: Table S5). All BPP analyses consistently supported group 5 (from southern China and Southeast Asia, recognized as *N. fulvescens*, Additional file 6: Table S6) and group 9 (identified as *N. huang*) as the same species. Group 8 was supported as a distinct putative species (putative sp. 3). Animals in this group were from Tibet, Yunnan, China, and northern Vietnam, and were morphologically similar to *N. fulvescens*, but did not have distinctively bicolored tails [5] (Additional file 7: Table S7).

**Table 4:** Results of Bayesian phylogenetics and phylogeography (BPP) analyses using coalescent species trees as guard trees

Mitochondrial phylogroups	5, 9	8, 5 + 9	10, 12	13, 14	24, 27	23, 30	29, 23 + 30	22, 23+ 29 + 30	18, 19	18 + 19, 22+ 29 + 23 + 30	21, 18 + 19 + 22 + 29 + 23 + 30
MtDNA and nuclear Algorithm 0	0.21	1.0	1.0	1.0	1.0	0.74	0.99	0.99	0.05	1.0	1.0
MtDNA and nuclearAlgorithm 1	0.21	1.0	1.0	1.0	1.0	0.74	0.99	1.0	0.05	1.0	1.0
nuclear Algorithm 0	0.15	1.0	0.99	1.0	1.0	0.08	0.20	0.31	0.07	0.55	0.71
nuclear Algorithm 1	0.19	1.0	0.99	1.0	1.0	0.08	0.26	0.32	0.05	0.52	0.65

When nuclear genes alone were used, BPP supported *N. coninga* (group 21), *N. lotipes* (group 23) and several populations of *N. confucianus* as one species (Fig. 5; Table 4; Additional file 5: Table S5). All analyses supported group 24 from western Yunnan and southeastern Tibet (identified as *N. confucianus mentosus*); group 26 from Gongbu, Tibet (identified as *N. confucianus*; putative sp. 4); and group 27 from southern China and northern Vietnam (identified as *N. confucianus*; putative sp. 5) as three putative species (Fig. 5; Table 4; Additional file 5: Table S5).

## Discussion

### Correct naming of species

According to the famous Chinese teacher Confucius, “If names be not correct, language is not in accordance with the truth of things. If language be not in accordance with the truth of things, affairs cannot be carried on to success” [22]. The species *N. confucianus* was named after Confucius [23], and his quote regarding names is indeed relevant to the taxonomy of the genus *Niviventer*. Species diversity within this genus has been underestimated [13, 14], and our understanding of its taxonomy was incomplete [9]. These two issues have prevented a full understanding of the systematics and evolution of the genus. Multi-locus sequencing for topotype, conducted in this study, indicates the need for several taxonomic and systematic revisions.

### Implications of the molecular species delimitation analyses

Based on the barcoding gap found from the pairwise distances of *CYT B* sequences, the single gene species delimitation method, ABGD, supported the existence of 22 groups/putative species in China (represented by 589 sequences from China), in agreement with previous reports by He and Jiang [13] and Lu et al. [14]. Although far fewer samples (*n* = 147) were available for multilocus species delimitation, BPP analyses still recognized 15 (using nuclear plus mitochondrial genes) or 11 (using three nuclear genes alone) putative species.

BPP molecular species delimitation approaches have been developed and widely used in mammals [24, 25], amphibians [26, 27], and reptiles [28, 29]. It should be noted that the biological species concept associated with BPP may not be true in practice [30], and that simulations showed that BPP is robust in the case of incomplete lineage sorting but sensitive to hybridization. Strongly supported putative species could be either valid species or long-term isolated conspecific populations. On the other hand, when named species were not strongly supported (PP < 0.95) as putative species by BPP, the results either suggested genetic homogenization (i.e., conspecific relationships) or gene flow between valid species. In both cases, the taxonomic status must still be evaluated carefully in a taxonomic context and must not violate the International Code of Zoological Nomenclature [31]. The BPP analysis that used nuclear genes alone did not strongly support *N. huang, N. lotipes*, or *N. coninga* as putative species. On the other hand, four putative new species (*N. confucianus mentosus* and *Niviventer* sp. 3–5) need further attention. Comprehensive morphological and morphometric examinations are warranted to test species status for all these taxa.

### Value of topotypical samples

Species in the genus *Niviventer* are morphologically very similar. The current taxonomy roughly relies on the size of skull, the length of ear and tail, and the color of skin and feet [3]. Morphological identification is not only time-consuming but also challenging. All but one of the topotypes of *Niviventer* sequenced in the current study were presented for the first time. We carefully assigned each individual to recognized species on the basis of morphology (Suppl. File 2) and the molecular results. These voucher specimens and sequences are valuable for barcoding-based species identification and taxonomic revision. As shown in our *CYT B* gene tree, GenBank sequences under the same species names were found in different groups or major clades (Fig. 1), indicating misidentification.

### Implications for taxonomy

The analyses of topotype sequences of named synonyms and subspecies revealed multiple putative species within the currently recognized species of *N. confucianus*, *N. fulvescens*, *N. andersoni*, and *N. eha*. Within *N. confucianus*, the subspecies *N. confucianus mentosus* should be examined more closely, as it was supported as a distinct group/putative species in all analyses. This subspecies was named and described as a species by Thomas [32] on the basis of the holotype from Hkampti, Myanmar, after which it was assigned as a subspecies of *N. niviventer* [33] or a synonym of *N. confucianus* [3]. Animals from Chayu (Tibet Autonomous Region), and Nujiang (Yunnan Province) were assigned to *N. c. mentosus* on the basis of their longer hind feet (≥30 mm) and white-tipped tail [34]. *Niviventer* sp. 4 collected from Guangxi and Yunnan were marked by a white tail tip and conspicuous brush, a typical pattern of *N. confucianus* that distinguishes it from *N. c. yaoshanensis*, *N. niviventer*, or *N. fulvescens. Niviventer* sp. 3 is similar to *N. fulvescens* in body size and color pattern but could be distinguished from *N. fulvescens* and *N. cremoriventer* by its fuzzy bicolor tail.

Our analyses also suggest that diversity in some groups may be overestimated. *Niviventer lotipes* (group 23), recognized by Li et al. [10] for its distinct karyotype, was not supported as a putative species by any of our BPP analyses. It was originally described as a subspecies of *N. confucianus* [35] on the basis of the holotype from Nada, Hainan, then treated as a subspecies of *N. niviventer* [33, 36] or a synonym of *N. tenaster* [9]. We did not included any topotype of *N. lotipes* (Danzhou, Hainan), but all specimens we included from Hainan, Fujian and Guangxi could be affiliated with the holotype (AMNH M-59303) of *N. lotipes* based on the sharp bicolor appearance of the tail, and are different from specimens used in Li et al. [10], showing a sharp bicolor appearance. However, individuals from other localities had a white-tipped tail. Another recognized species, *N. coninga* (group 21), was not supported by the BPP analyses using nuclear genes. Furthermore, most recognized subspecies of *N. confucianus* were clustered into two lineages or groups (subspecies *N. c. yushuensis*, *N. c. yajiangensis*, and *N. c. deqinensis* in group 29, and the nominated subspecies *N. c. confucianus*, *N. c. chihliensis*, and *N. c. naoniuensis* in group 30). They were not supported as putative species by BPP analyses. Therefore, the taxonomic status of all these clades must be re-examined.

## Conclusions

In this study, we collected extensive samples of *Niviventer* in China, including most topotypes of species, subspecies, and synonyms. We developed a robust phylogeny on the basis of one mitochondrial and three nuclear genes. Four major clades were recovered by both concatenation and coalescent-based approaches. The sister relationship between *N. brahma* and *N. eha* was well supported, and relationships among *N. confucianus*-like species were mostly well resolved. Our coalescent-based species delimitation analyses indicated that species diversity of *Niviventer* may be overestimated by single locus in previous studies, and three species should be re-evaluated using morphological or morphometric approaches. However, our analyses also indicated that cryptic or putative new species may exist in the mountains of southwest China, an area characterized by extremely complex topography, climate conditions, and geographic history.

## Methods

### Collection of samples and sequences

We collected 157 individuals of *Niviventer*, as well as five *Chiromyscus langbianis* (previously included in *Niviventer*), from China and neighboring northern Vietnam (Additional file 1: Table S1). These samples were collected at 67 locations (Fig. 1), including the type localities of 6 species, 8 subspecies, and 4 synonyms (Table 1). Voucher specimens from mainland China were deposited in the Kunming Institute of Zoology, Chinese Academy of Sciences, and Sichuan Academy of Forestry, China, and voucher specimens from Taiwan were deposited in Tunghai University. Specimens were carefully identified on the basis of external and skull characters following the original descriptions (Additional file 6: Table S6, [3, 5, 6]) and in consideration of the results of molecular phylogenetic analyses (see Results section). We followed the taxonomy of Musser and Carleton [9] but recognized *N. huang*, *N. bukit*, and *N. lotipes* as valid species [10, 11], and treated *N. langbianis* as a member of *Chiromyscus* following Balakirev et al. [12]. After completing our mitochondrial phylogeny, we carefully examined and described the morphological characters of each clade (Additional file 7: Table S7).

Total genomic DNA was extracted using the sodium dodecyl sulfate (SDS) method [37]. The complete mitochondrial cytochrome b (*CYT B*) gene and three nuclear gene segments (Exon 10 of the growth hormone receptor [*GHR*], Inter-photoreceptor retinoid binding protein [*IRBP*], and Recombination activation gene 1 [*RAG1*]) were amplified. Primers and annealing temperatures were either taken from the literature [38–40] or designed for this study (Table 5). PCR products were purified and sequenced in both directions using the BigDye Terminator Cycle kit v. 3.1 (Invitrogen, USA) on an ABI 3730xl sequencer (Applied Biosystems, USA).

**Table 5:** Primers and polymerase chain reaction (PCR) cycling conditions used in this study

Genes	Primer Name	Nucleotide sequence 5′ to 3′	Annealing Temperature (°C)	Fragment Length (bp)	Citation
*CYT B*	L14724_hk3	GGACTTATGACATGAAAAATCATCGTTG	47.5 °C	1140	He et al. 2010 [38]
	H15915_hk3	TCTCCATTTCTGGTTTACAAGAC			
*IRBP*	IRBP-217	ATCCCCTATGTCATCTCCTACYTG	60 °C	1149	Stanhope et al. 1992 [39]
	IRBP-1531	CGCAGGTCCATGATGAGGTGCTCCGTGTCCTG			
*RAG1*	S70	TCCGAGTGGAAATTTAAGMTGTT	51.7 °C	1221	Steppan et al. 2004 [40]
	S73	GAGGAAGGTRTTGACACGGATG			
*GHR*	GHR-F	GAGTTCATTGAGCTGGATAT	60 °C	777	Current Study
	GHR-R	ATGAGTTGCGCTGACGA			

We assembled and edited sequences using DNASTAR Lasergene v. 7.1 and aligned each gene using MUSCLE [41]. MEGA 7 was used for visual inspection [20].

### Phylogenetic reconstruction

Three data sets were used for phylogenetic analyses: 1) the *CYT B* data set, which included 674 in-group sequences (157 sequences from the current study and 517 sequences downloaded from GenBank; Table 2), representing 16 of 19 recognized *Niviventer* species and two potential new species identified in a previous study (namely, *Niviventer* sp. 1 and sp. 2) [18], with *Rattus andamanensis* and *Chiromyscus langbianis* as the outgroup. The first 60 bp and the last 63 bp of *CYT B* were deleted from the data set to avoid sequencing errors [13]. 2) A four-gene concatenated data set, including sequences of 197 individuals of *Niviventer* plus outgroups (157 sequences from the current study and 40 sequences downloaded from NCBI GenBank; Tables 2). One to four sequences downloaded from GenBank were selected as representative of each group based on the *CYT B* gene tree. 3) The full data set, including 147 sequences from the current study plus 8 individuals representing six outgroup species (*C. langbianis*, *L. edwardsi*, *L. neilli*, *R. andamanensis*, *R. norvegicus*, and *M. musculus*), all of which have four gene segments (*CYT B*, *GHR*, *IRBP*, and *RAG*1).

PartitionFinder v. 1.0.0 [19] was used to estimate the best-fit partitioning schemes and substitution evolutionary models under the Bayesian Information Criterion [42, 43]. For concatenated gene tree/species tree analyses, we concatenated gene alignments and defined by genes and codon positions. We did not consider the parameter for the proportion of invariant sites suggested by the author of RAxML [44]. Thus, we considered 12 models for each MrBayes and BEAST. For RAxML analyses, only GTR + gamma models were considered because RAxML did not allow any other models. For coalescent species tree estimation (see below), each of the three nuclear genes was treated as one partition and the *CYT B* was partitioned by codon positions. jModelTest 2 was used to estimate the best-fit evolutionary model for each nuclear gene [45].

The phylogenetic relationships of *Niviventer* were reconstructed using Bayesian inference and ML approaches. ML analyses and Bayesian analyses were conducted for the *CYT B* data set and the full data set, respectively, and both ML and Bayesian analyses were used for the concatenated data set. The ML analyses were performed using RAxML v7.2.8 on the CIPRES Science Gateway v 3.3 [46] (http://www.phylo.org). The BS were obtained using a rapid bootstrapping algorithm with 500 bootstrap replicates [44]. Bayesian analyses of the concatenated data set were implemented in MrBayes v. 3.2 and repeated twice [47]. Each analysis consisted of two independent runs, using four chains, sampled every 1,000 generations. We used Tracer v. 1.6 [48] to access the convergence of the two independent runs and stationary state of each parameter, and ESSs higher than 200 were considered adequate. Finally, each analysis was run for 2 million generations, and the first 25% of the generations were discarded as burn-in. Bayesian analyses of the full data set were implemented in BEAST v. 1.8.2 [48]. Each BEAST analysis used a lognormal relaxed clock model, a birth-death tree prior [49] and the other default parameters. The analyses were repeated twice; each was run for 200 million generations, and we sampled every 20,000 interactions. We considered BS ≥ 70 and PP ≥ 0.95 as strong support [50, 51].

### Species delimitation and species tree estimation

We used ABGD [21] to recover candidate species, and species tree and Bayesian Phylogenetics and Phylogeography (BPP v. 3.1; [52]) for validation. The ABGD approach assumed that the largest intraspecific divergence is still less than the smallest interspecies divergence. The gap between intra- and interspecific distance observed from the distribution of sequence distances is defined as a barcoding gap. ABGD automatically clusters the sequences into groups or hypothetical species based on the barcoding gap. Thus, detection of pairwise differences is the premise of this method. The only input parameter, although imprecisely, was the maximum prior intraspecific distance, *p*. We chose ABGD instead of bPTP [53] or GMYC [54] because this method was independent of phylogenetic trees and particularly sensitive to recent speciation events [55]. This analysis was performed on the ABGD website (http://wwwabi.snv.jussieu.fr/public/abgd/abgdweb.html) and used the 674 *CYT B* sequences as input data (Table 2). We selected the K80 model and set the transition/transversion ratio at 4.73, as estimated using MEGA 7 [20]. We set both the number of steps and bins to 25 and ran the program with different gap widths as 0.5, 1.0, 1.5, 2.0 and 2.5. Genetic distances of *CYT B* between candidate species were calculated using MEGA 7 [20] under the K2P model.

We inferred the species tree using the *BEAST model [56] implemented in BEAST v. 1.8.2. Species with at least one sequence per gene were included (as required by the model). We assigned the full data set of 147 individuals to 18 candidate species groups according to the results of ABGD species delimitation analyses (Additional file 1: Table S1). We run the analyses in BEAST v. 1.8.2 [48] for 400 million generations with the same priors as in the BEAST analyses.

To validate our ABGD proposal, a multilocus coalescent species delimitation method, BPP, was performed. Based on the phylogeny, ancestral population size, and root age, BPP employed the Reversible-Jump Markov Chain Monte Carlo (rjMCMC) method to generate the posterior probability that the two groups form a single species. Two data sets were used in these analyses: i) mitochondrial and nuclear genes and ii) three nuclear genes alone. Individuals represented only by mitochondrial sequences (sequences downloaded from GenBank) were not included in our BPP analyses. Using the results of AGBD, we assigned 147 individuals to 18 candidate species (Additional file 1: Table S1). We ran BPP for each of the four major clades recovered in our phylogenetic analyses separately (see Results), and used the *BEAST coalescent species tree as the guide tree. We performed the analyses using both rjMCMC algorithms 0 and 1. Analyses were run for one million generations with a pre-burn-in of 10,000 generations and a sampling interval of 100. We repeated BPP analyses 12 times with different combinations of priors and parameters following Wan et al. [25].

## Additional files

Additional file 1: Table S1.
*Niviventer* specimens collected for the present study. (XLSX 28 kb)
Additional file 2:Table S2. Sequence information downloaded from GenBank and used as outgroups. (XLSX 60 kb)
Additional file 3: Table S3. The best-fit partitioning schemes and evolutionary models estimated using PartitionFinder. (DOCX 17 kb)
Additional file 4: Table S4. Results of K2P genetic distance of *CYT B* between groups. (XLS 37 kb)
Additional file 5: Table S5. Algorithms, priors, and parameters for all Bayesian phylogenetics and phylogeography (BPP) analyses and results. (XLSX 16 kb)
Additional file 6: Table S6. Original description and a key to currently recognized species distributed in China. (DOCX 28 kb)
Additional file 7: Table S7. Morphological descriptions of each mitochondrial clade. (DOCX 56 kb)
