# Supplementary material for: Multi-locus phylogeny using topotype specimens sheds light on the systematics of Niviventer (Rodentia, Muridae) in China
Source: BMC Evol Biol. 2016 Dec 1;16:261. doi: 10.1186/s12862-016-0832-8 (PMC5133754; doi:10.1186/s12862-016-0832-8)
Supplement: Additional file 3: Table S3. — The best-fit partitioning schemes and evolutionary models estimated using PartitionFinder. (DOCX 17 kb) [file 12862_2016_832_MOESM3_ESM.docx]

| The best-fit partitioning scheme and evolutionary models for each analysis | | |
| --- | --- | --- |
|  | Best-fit partitioning scheme | Substitution evolutionary model |
| *CYT B* RAxML analyses | 1st codon = 1-1140\3; | GTR+G |
|  | 2nd codon = 2-1140\3; | GTR+G |
|  | 3rd codon = 3-1140\3; | GTR+G |
| Concatenated MrBayes analysis | p1 = 1-1140\3, 1143-1908\3; | SYM+G |
|  | p2 = 2-1140\3; | HKY+G |
|  | p3 = 3-1140\3; | GTR+G |
|  | p4 = 1141-1908\3, 1909-3057\3, 3058-4278\3; | HKY+G |
|  | p5 = 1142-1908\3, 1910-3057\3, 3059-4278\3; | HKY+G |
|  | p6 = 1911-3057\3, 3060-4278\3; | HKY+G |
| Concatenated RAxML analysis | p1 = 1-1140\3 | GTR+G |
|  | p2 = 2-1140\3, 1142-1908\3, 1910-3057\3, 3059-4278\3 | GTR+G |
|  | p3 = 3-1140\3 | GTR+G |
|  | p4 = 1141-1908\3, 1909-3057\3, 3058-4278\3 | GTR+G |
|  | p5 = 1143-1908\3, 1911-3057\3, 3060-4278\3 | GTR+G |
| Concatneated BEAST analysis | p1 = 1-1140\3; | TrNef+G |
|  | p2 = 2-1140\3; | HKY+G |
|  | p3 = 3-1140\3; | TrN+G |
|  | p4 = 1141-1908\3, 1909-3057\3, 3058-4278\3; | HKY+G |
|  | p5 = 1142-1908\3, 1910-3057\3, 3059-4278\3; | HKY+G |
|  | P6 = 1143-1908\3; | K80+G |
|  | P7 = 1911-3057\3, 3060-4278\3; | HKY+G |
| *BEAST analysis | CYTB 1st codon = 1-1140\3; | TrNef+G |
|  | CYTB 2nd codon = 2-1140\3; | HKY+G |
|  | CYTB 3rd codon = 3-1140\3; | TrN+G |
|  | GHR | K80+G |
|  | IRBP | HKY+G |
|  | RAG1 | K80+G |
