# Supplementary material for: Multi-locus phylogeny using topotype specimens sheds light on the systematics of Niviventer (Rodentia, Muridae) in China
Source: BMC Evol Biol. 2016 Dec 1;16:261. doi: 10.1186/s12862-016-0832-8 (PMC5133754; doi:10.1186/s12862-016-0832-8)
Supplement: Additional file 6: Table S6. — Original description and a key to currently recognized species distributed in China. (DOCX 28 kb) [file 12862_2016_832_MOESM6_ESM.docx]

|  | Species | Description | Diagnose |
| --- | --- | --- | --- |
| **1** | ***N. andersoni* (Thomas, 1911)** | Size large, fur coarse, not spinous.  Dorsal dull-lined clay, more buffy on the sides.  Ventral white.  Top of muzzle greyish, dark brown sides, this color passing backwards and joining the dark eye-rings.  Ears medium length, dull blackish.  Hands white, with a little dark on the metacarpus; feet brown over the greater part of metatarsus; toes and border of metatarsus white.  Tail very long, fairly well haired, not conspicuously tufted, above brown for proximal half, then changing gradually to white, below white except base.  Mammae 2—2 = 8.  Skull large, brain-case rather less convex above. | Endemic to southwest China. Like *N. excelsior*, but larger body and skull size, longer tail, more developed supraorbital ridge. They distinguished from the other *niviventer* by the combination of large body size, soft pelage without spines, longer tail with 1/4–1/2 white distal tip, the sides of the braincase slope outward from the dorsolateral margins to the squamosal roots, inconspicuous or absent of low ridges along edges of braincase (Musser, 1981). |
| **2** | ***N. brahma* (Thomas, 1914)** | Size large, fur woolly, soft, not spinous.  Dorsal cinnamon-brown, much buffy on the sides.  Ventral greyish white. White on throat and middle line of belly and chest.  Muzzle greyish, eyes surrounded by an obscurely darker ring.  Ears of medium size, naked, grey.  Hands and feet brown over the metacarpus and metatarsus, the edges and digits white; pads large; fifth hind toe long, reaching to the middle of the second phalanx of the fourth.  Tail long, well haired, slightly tufted; dark brown above, rather lighter below.  Mammae 1—2 = 6.  Skull light and thinly. Muzzle and interorbital region narrow. Developed supraorbital ridge but dying off on parietals. Brain-case smooth, rounded, convex above. Palatal foramina well open, extending backwards to the level of the front root of M^1^. Bullae small. | Similar to *N. eha*, but larger size and stouter skull, more developed supraorbital and temporal ridges. Both different from the other *niviventer* in soft fur without spines, gray belly, three mammae, long and tufted tails, and zygomatic plates not produced forward, nearly vertical. |
| **3** | ***N. confucianus* (Milne-Edwards, 1871)** | Small size, fur soft with seasonal spines.  Dorsal brown and black, dull tawny hue on the sides.  Ventral white, with a faint sulphury suffusion.  Muzzle brown.  Ear large and thin, blackish brown.  Hands Dusky over the center of the metacarpal area and the basal two-thirds of the metatarsals, and base of digits 2/5, dark brownish.  Tail slightly longer than head and body, well haired and tufted, above blackish, below usually more or less of its tip all around, white.(Milne-Edwards et al., 1868)  Mammae 2—2 = 8.  Skull stocky, with large bullae (Allen, 1940). | Resembling *N. fulvescens*, but larger body size, darker color, greater ear, frequently shorter white-tipped tail, longer nasal, heavier molars and, larger and more globose bullae (Osgood, 1932). |
| **4** | ***N. coninga* (Swinhoe, 1864)** | Large size, Fur densely spinous.  Above reddish brown, with stiff black bristles.  Below white.  Buff on each side of the muzzle at the bases of the vibrissae, eye surrounded by a black ring.  Ear and fore part of legs deep brown.  Back of hands and feet brown, toes white.  Tail long, scantily haired, tufted, above 3/4 deep brown, below and terminal 1.75 inch all around, white.  Mammae 2—2 = 8.  Skull large, zygomatic plates project well in front of the arches, prominent ridges outlining the area behind the interorbital area and edges of the braincase. | Endemic to Taiwan. With much appearance of *N. culturatus*, but reddish upperpart, white belly, larger body size, white hands, greater skull, larger and longer molar row and border zygomatic board. Different from *N. andersoni* and *N. excelsior* in spinous fur, vertical braincase sides, and more developed temporal ridges outline parietal. |
| **5** | ***N. culturatus* (Thomas, 1917)** | Large size, fur long and shaggy.  Above from mouse gray to hair brown；  Below creamy white.  Hands white.  Feet grayish brown with digits white.  Tail long, well haired, with tufted, above blackish for three-fourths, below and terminal all around, white.  Mammae 1—1—2 = 8.  Skull large, with well-developed supraorbital ridges, long palatal foramina. | Endemic to Taiwan, Appearance about as *N. excelsior*, but darker and spinous, cream white belly, more developed supraorbital and temporal ridges and vertical braincase sides. Different from *N. coninga* in darker dorsal, smaller body and skull size, short teeth row. Different from other by large body size and 1/4–1/3 white tip tail. |
| **6** | ***N. eha* (Wroughton, 1916)** | Small size, fur soft, without spines.  Above bright clay-color, darker and duller in the mid-dorsal.  Below grayish white.  Muzzle black, meeting with the black eye-ring forming a mask.  Hands and feet dark, toes white.  Tail much long, with short black hair, above dark, below white.  Mammae 1—2 = 6.  Skull small, with undeveloped supraorbital ridges, small teeth. Zygomatic plates not produced forward, not visible from above. | Near *N. brahma*, but smaller body and skull size, fragile skull without ridges along the edges of interorbital and braincase. They different from other *niviventer* in gray belly, three pair mammae and nearly vertical zygomatic plates. |
| **7** | ***N. excelsior* (Thomas, 1911)** | Large size, fur long and coarse, without spines.  Above cinnamon or clay-color, more buffy on the sides.  Below white or creamy white.  Muzzle greyer, dark eye-ring.  Ear medium length, greyish brown.  Hands white, feet with dark metatarsal patches.  Tail rather long, well haired, with tufted, above brown for two-thirds, below white and the terminal all around.  Mammae 2—2 = 8.  Skull large, Nasals long and slender, Interorbital region narrow, its borders sharp-edged, but without definite beads. Brain-case smooth, markedly convex above. Anteorbital plate little projected. Palatal foramen long. Bullae small. | Endemic to southwest China. Like *N. andersoni*, but smaller body and skull size, fragile skull and undeveloped interorbital ridges. They distinguished from the other *niviventer* by the combination of large body size, soft pelage, long tail with 1/4–1/2 white distal tip, sloping braincase sides, inconspicuous or absent of low temporal ridges. |
| **8** | ***N. fulvescens* (Gray, 1847)** | Medium size, fur spinous.  Above a bright fox color, black in the mid-dorsal area, clear on sides.  Below white.  Muzzle duller brownish.  Ear small, brown.  Hands and feet have a narrow dusk median area mixed with buffy, and passing into clear buff, the white, at the sides of the feet.  Tail slender and long, root and above fulvous, then bicolor, below white.  Mammae 2—2 = 8.  Skull delicate and slender, low, flatten, ridges prominent in orbit area, then less, rostrum long and slender (Allen, 1940). | Similar to *N. niviventer*, but brownish pelage, longer tail with dark brown band up to 10 mm (Musser, 1973), narrow and deep skull (Abe, 1977). Different from other *niviventer* in brighter color, spinous fur, longer bicolor tail, slender skull and smaller bullae. |
| **9** | ***N. huang* (Bonhote, 1905)** | Medium size, fur with black spines.  Above ochraceous-rufous, darker along the dorsal area, much rufous on the sides.  Below white.  Ears moderately long and sparsely covered with very close, short, dark brown hairs.  The feet are whitish, with the rufous color running down the centre of their upper surface.  Tail rather longer than the head and body, clothed with short hairs and bicolor.  Mammae 2—2 = 8.  The skull medium size, the supraorbital ridges being continued right across to the posterior margin of the parietal. | Similar to *N. fulvescens*, but pelage brighter, slightly smaller and shorter-tailed, a reduction or absence of dusky markings on the feet (Osgood, 1932). |
| **10** | ***Chiromyscus. langbianis*** (Robinson and Kloss, 1922) | Medium size, fur soft and dense with spines.  Above brownish gray suffused with pale yellowish orange, with olive-gray tone, brighter on the sides.  Below white.  Ear large (short, (Balakirev et al., 2014).  Hands and feet wide, with broad brown or chestnut stripe, fingers white (Balakirev et al., 2014).  Mammae 2—2 = 8.  Tail long, well haired, tufted (Balakirev et al., 2014), dark brown throughout (Musser, 1973).  Skull rostrum shorter relative to length of skull and more rectangular, ridges developed, | Similar to *N. cremoriventer*, but a little duller in color, longer feet, larger ears, tail more finely ringed, short nasals, shorter and narrower rostrum, larger and more inflated bullae, longer incisive foramina, heavier and higher ridges and wider braincase (Musser, 1973). Both different from other *niviventer* in dark brown tail. |
| **11** | ***N. lotipes*** (Allen, 1926) | Medium size, fur with spines.  Dorsal mixed ochraceous and black, the sides less black.  Below slightly sulphury white.  Back of hands and feet white, lacking all trace of the dark area on the central area.  Muzzle ochraceous.  Tail less thickly haired, sharply bicolor tail, dark above, white throughout below.  Mammae 2—2 = 8.  Skull, compared with typical *confucianus* the skull of the Hainan race presents no special peculiarities, except that in adults it appears slightly heavier, or may reach a slightly greater average size. | Similar to *N. confucianus*, but different in brighter, more ochraceous, spinous, and sulphury belly, white feet and bicolor tail without white tip. |

Reference

Abe, H., 1977. Variation and taxonomy of some small mammals from central Nepal. J. Mammal. Soc. Japan, 7, 63–73.

Allen, G.M., 1926. Rats (genus *Rattus*) from the Asiatic Expeditions. Amer. Mus. novitates. no. 217, 1–16.

Allen, G.M., 1940. The Mammals of China and Mongolia: Natural history of Central Asia V Π. Walter Granger, ed. American Museum of Natural History.

Balakirev, A.E., Abramov, A.V., Rozhnov, V.V., 2014. Phylogenetic relationships in the *Niviventer*-*Chiromyscus* complex (Rodentia, Muridae) inferred from molecular data, with description of a new species. Zookeys, 109–136.

Bonhote, J.L., 1905. The Mammalian Family of China.Part I. Proc. Zool. Soc. London 2, 384–397.

Gray, 1846. Catalogue of the specimens and drawings of mammalia and birds of Nepal and Thibet. Presented by B.H. Hodgson to the British Museum. By order of the Trustees, London.

Milne-Edwards, H., 1871. Nouv. Arch. Mus. d'Hist. Nat. Paris.

Milne-Edwards, H., Huet, Louveau, Mesnel, A., Milne-Edwards, A., Severeyns, G., Imp. Becquet, Imprimerie de É. Martinet, 1868. Recherches pour servir à l'histoire naturelle des mammifères: comprenant des considérations sur la classification de ces animaux. G. Masson, Paris.

Musser, G.G., 1973. Species-limits of *Rattus Cremoriventer* and *Rattus Langbianis*, Murid Rodents of Southeast Asia and the Greater Sunda Islands. Am. Mus. Novit. 2525, 1–65.

Musser, G.G., 1981. Notes on systematics of Indo-Malayan murid rodents, and descriptions of new genera and species from Ceylon, Sulawesi, and the Philippines. Bull. Am. Mus. Nat. Hist. 168, 229–330.

Osgood, W.H., 1932. Mammals of the Kelley-Roosevelts and Delacour Asiatic expedition, Chicago.

Robinson, H.C., Kloss, C.B., 1922. New mammals from French Indo-China and Siam. Ann. Mag. Nat. Hist. 9, 87–99.

Swinhoe, R., 1864. On a New Rat from Formosa. Proc. Zool. Soc. London, 185–187.

Thomas, O., 1911. The Duke of Bedford’ s Zoological Exploration of Eastern Asia.— XIII. On Mammals from the Provinces of Kan-su and Sze-chwan, Western China. Proc. Zool. Soc. London 81, 158–180.

Thomas, O., 1914. On small mammals collected in Tibet and the Mishmi Hills. J. Bombay Nat. Hist. Soc. 23, 230–233.

Thomas, O., 1917. Two new Rats of the *Rattus confucianu*s Group. Ann. Mag. Nat. Hist. 20, 198–200.

Wroughton, R.C., 1916. New Rodents from Sikkim. J. Bombay Nat. Hist. Soc. 24, 424–430.

Identification Key to the species of *Niviventer* distributed in China

1 Only distributed in Taiwan 2

1’ Distributed in mainland 3

2 Dorsum reddish brown, densely spinous; Venter with prominent pectoral patches and sometimes midventral streaks; back of the hand and feed brown; tail dark above and white below to tip or terminal 1.75 inch white all around; greatest length of skull longer than 40mm ***N. coninga***

2’Dorsum from mouse gray to hair brown, without spines; Venter without prominent pectoral patches or midventral streaks; back of the hand and feed white; tail blackish for three-fourths above and white below and terminal all around; greatest length of skull shorter than 40mm ***N. culturatus***

3 Venter gray; three pairs of mammae 4

3’Venter white or cream; four pairs of mammae 5

4, Length of head and body longer than 134mm; without tuft at base of each ear; greatest length of skull longer than 35mm; supraorbital ridges well developed ***N. brahma***

4’ Length of head and body shorter than 134mm; with tuft at base of each ear; greatest length of skull longer than 35mm; supraorbital ridges absent ***N. eha***

5 Pelage without spines; the distal half, two-thirds, or one-fourth of the tails are white and prominently tufted; greatest length of skull longer than 38mm; length of upper moral tooth row usual longer than 7 mm; inconspicuous or absent low ridges along the dorsolateral edges of the braincase to the occiput; the sides of the braincase slope outward from the smooth dorsolateral margins to the squamosal roots 6

5’Pelage spinous or seasonal spinous; tail dark above and white below, from base to tip or with about 5cm white tip; greatest length of skull shorter than 38mm; length of upper moral tooth row usual shorter than 7 mm; low ridges along the dorsolateral edges of the braincase to the occiput or near so; the sides of the braincase vertical from the smooth dorsolateral margins to the squamosal roots 7

6 Dorsal coarse clay-color, more buffy on the sides, hairs about 10mm; length of upper moral tooth row usual longer than 7.2 mm; interorbital ridges well-developed ***N. andersoni***

6’ Dorsal coarse more bright ochraceous buff, hairs about 14mm; length of upper moral tooth row usual shorter than 7.2 mm; interorbital ridges absent ***N. excelsior***

7 Dorsum dark slate, brown; tail well haired, as well as or slightly longer than body and head, tufted; audial bullae larger and globose; the supraorbital ridges frequently ending in front of the posterior margin of the parietal 8

7’ Dorsum bright reddish or ochraceous; tail rarely haired, no tufted, 1.3-1.5 times length of body and head; audial bullae small and flat; the supraorbital ridges across to the posterior margin of the parietal 9

8 Distributed in most part of China; back of hind feet with dark brownish area; tail haired, frequently with white tip or spot on the upper side ***N. confucianus***

8’ Distributed in Hainan, China; back of hind feet white, without the darkened central area; tail less haired, bicolor from base to tip ***N. lotipes***

9 Dorsum bright fulvous, without dark along the dorsal area; the back of hind feet with narrow dusky area; tail length accounting for 58-59% of the total length ***N. fulvescens***

9’ Dorsum duller, more ochraceous, darker along the dorsal area; the back of hind feet white, with the rufous color running down the centre of their upper surface; tail length accounting for 54-57% of the total length ***N. huang***
