# Supplementary material for: Multi-locus phylogeny using topotype specimens sheds light on the systematics of Niviventer (Rodentia, Muridae) in China
Source: BMC Evol Biol. 2016 Dec 1;16:261. doi: 10.1186/s12862-016-0832-8 (PMC5133754; doi:10.1186/s12862-016-0832-8)
Supplement: Additional file 7: Table S7. — Morphological descriptions of each mitochondrial clade. (DOCX 56 kb) [file 12862_2016_832_MOESM7_ESM.docx]

Supplementary Table 3 Specimen description grouped in this study

| Group code | Description, comparison and identification | Observed specimens |
| --- | --- | --- |
| 5 | Animals in this lineage are relatively small (115-142 mm.). Dorsal and ventral pelage is sharply bicolored. The dorsal pelage is brightly ochraceous-orange in color, and thickly spinous, more yellow in the sides. The fur of the back and sides is composed of three kinds of hair, (a) soft underfur is slate at base and bright fulvous at tip; (b) Numerous stiff spines mixed with underfur, greenish horn color at base, those on the mid-dorsal area black at tip, and on sides uniform throughout; (c) longer bristles up to 14 mm. in length, blackish throughout, especial on mid-dorsal. Ears small (19-21 mm.), naked, gray brown. The ventral white throughout. Hands and hindfeet dorsal white, few with light brown streak over metacarpus or metatarsus area (n=1). Hindfeet medium (26-28 mm.). Tail much longer than head and body (130-139% of HB), short haired, bicolored from base to tip, Not tufted. Four pairs of mammae.  Skull slender. Supraorbital ridges well developed, much more curved backwards, and extend backwards to poster margin of parietals. The sides of the braincase are vertical from parietal edges to the squamosal roots. Zygomatic plates much forward. Nasals short (9.28-14.6 mm.). Upper molar medium big relative to skull. Incisive foramen short (5.23-6.44 mm.), most posterior ending before the level of the first moral. Brain case convex above. Bullae very small.  Most samples/specimens from Guangxi and southern Yunnan, China, and northmost Vietnam. To include samples from Thailand presented by Page et al. 2010 (R3427, R4723, R4525, R3459, R3429), and presented by Latinne et al. 2013 (HCBNF01, HCBNF02, HCBNF03, HCBNF04, HCBNF05), in which identified as *N. fulvescens*. However samples from Vietnam presented by Balakirev et al., 2010 (K-44, K-59, K-60, LO-4, LO-8, LO-9) were identified as *N. huang*.  The specimens in this group differs from *N. brahma* and *N. eha* with white belly, from *N. andersoni*, *N. excelsior*, *N. culturatus* and *N. coninga* with the small body size, and from *N. confucianus*, *N. lotipes* with the short bicolor tail. Those specimens in this group fit best with the description of *N. fulvescens* [[1](#_ENREF_1), [2](#_ENREF_2)], comparing with the holotype of *N. huang vulpicolor* (AMNH43297), *N. gracilis* (AMNH101520)*, N. fulvescens lepidus* (AMNH104127)*,* the synonyms of *N. fulvescens* which we observed. | 0812130, 0812133, BN1409076, Z201401502, Z201401503. |
| 8 | Most animals in this lineage are medium size (127-151 mm.). Dorsal and ventral pelage is sharply bicolored. The dorsal pelage is nut brown-yellow, shading blackish in the mid-dorsal area, and most thickly spinous (n=4). The fur of the back and sides is composed of three kinds of hair, (a) soft underfur is slate at base and brown-yellow at tip; (b) Flattened grooved spines are sand yellow at base, those on the mid-dorsal area black at tip, and on sides uniform throughout; (c) bristles are black throughout, up to 18mm. in length, dominating in the mid-dorsal area. Ears small length (18-21 mm.), naked, light brown. The ventral white throughout, some with a dark brow patch on the chest (n=3), and some with white stiff spines. Pattern of hands and hind feet back is variable, with a light brown streak (n=3), white (n=1) or dark yellow (n=2). Hind feet long (26.5-30 mm.). Tail slender and much longer than head and body (130-181% of HB), short haired, indistinct bicolored, dark above, paler below but only about 1/3 of the area of tail surface, with black tufted. Four pairs of mammae.  Skull similar group 5, slightly larger in size, Incisive foramen longer (5.52-7.28 mm.), most posterior ending backward beyond the level of the first moral, and wider.  Most samples/specimens from southern Yunnan, China. To include samples from Vietnam presented by  Balakirev et al., 2010 (SH-9, SH-11, SH-13, SH-14, SH-22, SH-25, SH-59, SH-127, SH-157, SH-164), and from Yunnan and Xizang, China presented by Lu et al. 2015 (XZ-ZM02M, XZ-ZM11, XZ-ZM12, XZ-ZM17, YN-LC090, YN-LC096, YN-LC108, YN-TC481), in which identified as *N. fulvescens*. However samples collected from central Yunnan, China presented by Jing et al., 2007 (wls11, wls22, wls23), in which identified as *N. cremoriventer*.  The specimens in this group can be distinguish from the other recognized *Niviventer* in the indistinct bicolored tail. In addition, those specimens are different from the holotype of *N. cremoriventer* (AMNH86770) and *N. cremoriventer baruss* (AMNH141208, synonym of *N. cremoriventer*`) in bigger body size, smaller ears and longer hind feet. Those specimens much similar to *N. fulvescens*, but different in the indistinct bicolored and tufted tail. | 0410124, 0812007, 20600, 0812001, 0812010, 0812093. |
| 9 | Most animals in this lineage are variable from small to medium size (127-151 mm.). Dorsal and ventral pelage is sharply bicolored. The dorsal pelage is ochraceous-orange mixed with blackish, and most thickly spinous (n=9). The fur of the back and sides is composed of three kinds of hair, (a) soft underfur is slate at base and yellow at tip; (b) Flattened grooved spines are pale white at base, those on the mid-dorsal area black at tip, and on sides uniform throughout; (c) bristles are black throughout, up to 20mm., dominating in the mid-dorsal area. Ears small length (19-21.5 mm.), naked, blackish. The ventral white throughout. Hands and hind feet white, some with light ochraceous tone or narrow black streak. Hind feet variable in length (26-30 mm.). Tail slender and longer than head and body (112-142% of HB), short haired, indistinct bicolored, dark above and white below from base to tip, without tufted. Four pairs of mammae.  Skull similar to group 5, no markedly difference.  Most samples/specimens from southern China, including Hainan. To include samples from Southern China presented by Lu et al. 2015, in which identified as *N. huang* or *N. ling*.  The specimens in this group are different from *N. andersoni*, *N. excelsior* in the sharply bicolor tail, from *N. brahma* and *N. eha* in the white belly, from *N. confucianus* and *N. lotipe* in the no tufted tail and ochraceous-orange pelage, and from *N. fulvescens* in the white hind feet different. Those specimens completely fit with the description of *N. huang* [[3](#_ENREF_3)]. | 0905261, 0905262, Z201312272, Z201312282, Z201312286, Z201312382, Z201312387, Z201312387, Z201312465, Z201312468, Z201312483, HN0003, HN0009, HN0010, HN0011, HN0015. |
| 10 | Animals in this lineage are variable from small to large (113-170 mm.), most medium (137.4±21.34). Dorsal and ventral pelage is bicolored. The dorsal pelage is fawn brown in color, not spinous, more yellow in the sides. The fur of the back and sides is composed of two kinds of hair, (a) soft underfur is slate gray at base and fulvous at tip; (b) slightly bristles up to 20 mm. in length, blackish throughout. Ears medium length (21.5-25 mm.), naked, gray brown. The ventral The ventral is gray white, slate gray same as underfur at base and white at tip, but hair white to their base at throat. Hands and feet are black over most part of metacarpus and metatarsus. Tail slender, and much longer than head and body (150-176% of HB), short haired, indistinct bicolored, dark above and below lighter, with distinct black tufted. Three pairs of mammae.  Skull large and slender. Supraorbital ridges well developed and extend backwards to middle of parietal. The sides of the braincase vertical from parietal edges to the squamosal roots. Zygomatic plates not produced forward, nearly vertical. Molar medium size. Incisive foramen well opened, their posterior ending extending backwards to the level of the first moral. Brain case convex above. Bullae very small.  Samples/specimens from Gaoligong Mountain Yunnan, China. To include samples from same area presented by Jing et al. 2007 (Glgs017), in which identified as *N. brahma*.  The gray belly and three pairs of mammae of the specimens in this group are unique characters of *N. brahma* and *N. eha*. But the large body size and developed inter-postorbital ridge can distinguished them from *N. eha*, fit best with the description of *N. brahma* [[4-7](#_ENREF_4)]. | PM1311181, PM13111193, PM1311297, LS1312145, LS1312863. |
| 12 | Similar to group 10, but Smaller in size (97-120 mm.), lighter dorsal color, shorter black bristles which only up to 15mm. in length, and ventral hair darker at base and throat. Tail distinct bicolored, dark above and below white throughout, with distinct black tufted. Three pairs of mammae.  Skull small and delicate. Supraorbital ridges undeveloped. The braincase is smooth, no ridges outlining edges of parietals. The sides of the braincase vertical from parietal edges to the squamosal roots. Zygomatic plates not produced forward, nearly vertical. Molars very small. Incisive foramen well opened, their posterior ending extending backwards to the level of the first moral. Braincase convex above. Bullae very small.  Samples/specimens from Gaoligong Mountain, Yunnan, China. To include samples from Deqin, Yunnan, China presented by Lu et al. 2015 (GZB225), in which identified as *N. eha*.  The gray belly and three pairs of mammae are unique characters of *N. brahma* and *N. eha*. But the small body size and inter-postorbital ridge absent can distinguished from *N. brahma*, fit best with the description of *N. eha* [[1](#_ENREF_1), [5](#_ENREF_5), [7-9](#_ENREF_7)]. | PM1312484, PM1312863, LS1312756, LS1312781 |
| 13 | Animals in this lineage are large (142-160 mm.). Dorsal and ventral pelage is bicolored. The dorsal pelage is clay brown in color, not spinous, shading black on the mid-dorsal and slightly bright in the sides. The fur of the back and sides is composed of two kinds of hair, (a) soft underfur is slate gray at base and clay brown at tip; (b) longer bristles up to 20 mm. in length, gray or white at base and black at tip. Ears medium (22-25mm.), naked, gray brown. The ventral white throughout, but the whitish was limited on inner side of hind legs. Hands and feet are black over centre of back. Tail much longer than head and body (152-169% of HB), well haired. Base of the tail is dark brown all around, white gradually trespassing on below to near half, and then white all around to tip with distinct white tufted. Four pairs of mammae.  Skull large. Supraorbital ridge undeveloped and no ridge outline the marge of parietals. The sides of the braincase slope outward from parietal edges to the squamosal roots. Zygomatic plates produced forward. Moral large and long. Incisive foramen short, their posterior ending in front or on level of the first moral. Bullae small relative to size of skull.  Most samples/specimens from Wester Sichuan, China. To include samples from Xizang and Sichuan, China presented Chen et al. 2010 and 2011 (XZC002, XZC004, XZC006, XZC007, XZC010, XZC012, SCSJ1, SCSH3, CWL52, BT003, YJ002), from Shangri-la, Yunnan, China by Lu et al. 2015 (YN-ZD188, YN-ZD227, YN-ZD266) and from Wester China by Jansa et al., 2006 (USNM 574372), in which identified as *N. excelsior*.  The specimens in this group differs from *N. brahma*, *N. eha*, *N. fulvescens*, *N. huang*, *N. lotipes* and *N. coninga* by white tipped tail, from *N. confucianus* *N. culturatus* and *N. andersoni* by the skull without interorbital ridge different. The specimens in this group fit best with the description of *N. excelsior* [[7](#_ENREF_7), [10](#_ENREF_10), [11](#_ENREF_11)]. | 0905210, Z201405692, Z201405693, Z201405694, Z201405699, Z201405703. |
| 14 | Similar to group 13, but larger in size, and grayer. Tail much longer than head and body (124-186% of HB), shorter distal tip, quarter to third. Four pairs of mammae.  Skull similar to group 13, but larger in size, more developed supraorbital ridge, larger moral and longer moral row.  Most samples/specimens from Yunnan, Sichuan and Shanxi, China. To include samples from Xizang and Sichuan, China presented Chen et al. 2010, 2011 and 2012 (JJSA019, JJSA020, XZC011, SCSD003, SCE032, SCSK3, SCSL007, JL003), in which identified as *N. andersoni*; and from Ailao Mountain and Gaoligong Mountain, Yunnan, China by Jing et al. 2007 (030, 04043, 070, 029, Glgs065, Glgs066), in which identified as *N. excelsior*; and from Yunnan, Sichuan and Hubei, China by Lu et al., 2015.  The specimens in this group differs from *N. brahma*, *N. eha*, *N. fulvescens*, *N. huang*, *N. lotipes* and *N. coninga* by the white tipped tail , from *N. excelsior* by the developed interorbital ridge, and from *N. confucianus* and *N. culturatus* by the undeveloped postorbital ridge. Those specimens fit best with the holotype of *N. a. ailaoshanensis* (KIZ99263), *N. a. pianmaensis* (KIZ74096) that we have observed [[7](#_ENREF_7), [10-12](#_ENREF_10)]. | 0612199, 0612198, Al1305314, 201211103, 0709240, H0281, H2044, PM1311382, PM1311524, 201309001, 0810274, Y204021, Z201405626, Z201405716, 0509084. |
| 18 | Animals in this lineage are small to medium in size (108-131 mm.). Dorsal and ventral pelage is bicolored. The dorsal pelage is gray brown in color, heavily spinous. The fur of the back and sides is composed of three kinds of hair, (a) soft underfur is slate gray at base and brown at tip; (b) Numerous flattened grooved spines are pale white throughout; (c) longer bristles up to 20 mm. in length, gray or white at base and black at tip. Ears large (27-29mm.), naked, brown. The ventral white throughout. Hands and feet are white or slightly brown trace over centre area. Tail slightly longer than head and body (117-139 % of HB), very well haired, above brown, white below and gradually trespassing on the above in distal third, with distinct black tufted. Four pairs of mammae.  Skull medium size and robust. Supraorbital ridges well developed and extend backwards to the notch of parietal. The sides of the braincase are vertical from parietal edges to the squamosal roots. Zygomatic plates produced forward. Molars medium. Incisive foramen well opened, their posterior ending extending backwards to the level of the first moral. Braincase slightly convex above. Bullae big and global.  Samples/specimens from Yantai, Shandong, China.  The specimens in this group differs from *N. brahma*, *N. eha*, *N. fulvescens*, *N. huang*, *N. lotipes* and *N. coninga* by white tipped tail, from *N. excelsior* by the developed interorbital ridge, from *N. andersoni* by the developed postorbital ridge and from *N. culturatus* by small body size and the white or slightly brown hind feet. Those specimens fit best with the description of *N. confucianus sacer* [[1](#_ENREF_1), [13](#_ENREF_13)]*.* | Z201311107, Z201311113, Z201311115, Z201311116, Z201311117 |
| 19 | Two specimens were observed in medium size (128, 166 mm.) in this group. Dorsal and ventral pelage is bicolored. The dorsal pelage is gray yellow in color, with spines. The fur of the back and sides is composed of three kinds of hair, (a) soft underfur is slate gray at base and pray yellow at tip; (b) smaller one with soft, and bigger one with thickly spines and expanding to venter. Spines are pale white throughout; (c) longer bristles up to 15 mm. in length, white at base and black at tip. Ears small (21, 22), naked, brown. The ventral white throughout. Hands and feet are white over centre area. Tail slightly longer than head and body (BN1409075, 120 % of HB), very well haired, above brown, above brown, white below and gradually trespassing on the above in distal third, no wholly white part with distinct black tufted. Four pairs of mammae.  Similar to group 18, but Supraorbital ridges extend backwards to poster margin of parietals and tail without white tip or brush.  Specimens in this group much similar to the description of *N. confucianus*. | 1001034, BN1409075 |
| 23 | Animals in this lineage are variable from small to medium in size (103-149 mm.). Color and pattern are similar to group 18, Ears small (19-22mm.), naked, brown. The ventral white throughout. Back of Hands and feet are white or with brown streak over centre area. Tail slightly longer than head and body (117-148 % of HB), short haired, above brown, white below, most bicolor throughout, few with white patch or part on dorsum, not conspicuously tufted. Four pairs of mammae.  Skull small in size. Supraorbital ridges low, not curved backwards, extending backwards to the notch of parietal. The sides of the braincase are near vertical from parietal edges to the squamosal roots. Zygomatic plates produced forward. Molars medium. Incisive foramen well opened, their posterior ending extending backwards beyond the level of the first moral. Braincase slightly flattened. Bullae big and global.  Most samples/specimens from southern China. To include samples from southern China including Hainan presented by Lu et al. 2012 and 2015, from Zhejiang presented by Wang et al., Unpublished, and from Xizang present by Chen et al., 2011, in which identified as *N. confucianus* or *N. lotipes*.  The specimens in this group differs from *N. brahma* and *N. eha* by the white belly. Samples from Guangxi, Fujian which differ from *N. fulvescens*, *N. huang*, *N. lotipes*, *N. coninga N. culturatus*, *N. andersoni* and *N. excelsior* by short and sharply bicolor tail and fit with the holotype of *N. lotipes* (AMNH59303) [[14](#_ENREF_14)], which different from the specimens of Li et al., 2008. The others fit best with *N. confucianus*. | LS13121185, H201301005, HS1302001, Y204047, Z201311182, Z201311200, Z201312258, Z201312268, Z201312288, Z201312348, Z201312359, Z201312422, Z201401501, Z201401505 |
| 24 | Animals in this lineage are variable from small to medium in size (115-169 mm.), most medium (139.71±21.09). Dorsal and ventral are sharply bicolored. The dorsal pelage is gray brown, more black in the mid-dorsal area, thickly spinous. The fur of the back and sides is composed of three kinds of hair, (a) soft underfur is slate gray at base and brown at tip; (b) Numerous flattened grooved spines are pale white throughout; (c) longer bristles up to 20 mm. in length, black throughout. Ears small to medium (18-23mm.), naked, light brown. The ventral white throughout. Hands and feet are white, brown over centre area. Hind feet longer (28-35, 31±2.16 mm).Tail slightly longer than head and body (103-137 % of HB), short haired, above brown, white below and gradually trespassing on the above in distal third, some forming white tail tip, no conspicuously tufted. Four pairs of mammae.  Skull similar to group 18, but bigger in size, stronger, supraorbital ridges low only extending backwards to the notch of parietal, not to the poster margin; longer rostrum, smaller and more flattened bullae.  Most samples/specimens from western Yunnan and Southeast Xizang, China. To include samples from Motuo, Xizang, China presented by Chen et al., 2011 (MT044, MT045, MT046), in which identified as *N. confucianus*.  The specimens in this group differ from *N. brahma*, *N. eha*, *N. fulvescens*, *N. huang*, *N. lotipes* *N. coninga*, *N. culturatus*, *N. andersoni* and *N. excelsior* by the short tai. The hind feet of those specimens longer than 30 mm fit with *N. confucianus mentosus* [[15](#_ENREF_15), [16](#_ENREF_16)]. | 0410011, GLGS6810, CBG07003, CBG07004, CBG07007, CBG07010, CBG07011 |
| 27 | Animals in this lineage are variable from small to medium in size (104-156 mm.), most small (128.86±16.66). Dorsal and ventral are sharply bicolored. Color and pattern similar to group 18, but more reddish, not spinous, shorter bristles (up to 18 mm.). Ears medium (21-25mm.), naked, light brown. The ventral white throughout. Hands and feet are white with a narrow brown streak over centre area. Tail longer than head and body (128-158 % of HB), slender, short haired and distal longer, above brown for proximal 2/3, and white below and distal third all around, no conspicuously tufted. Four pairs of mammae.  Skull variable, general two sizes, small and medium size. The smaller ones similar to group 23; and the bigger ones similar to group 24, but shorter rostrum, larger molars and bullae.  Most samples/specimens from Yunnan and Guangxi, China. To include samples from Yunnan, China presented by Lu et al., 2015 (YN-LC100, YN-LC119, YN-LC154, YN-TC280, YN-TC283, YN-TC471), and from Vietnam presented by Balakirev et al., 2010 (SH-16, SH-161), in which identified as *N. niviventer*; From Yunnan, China presented by Jing et al. 2007 (Wls001, Wls002, Wls005, 019, 069, Glgs034), in which identified as *N. fulvescens* or *N. eha* (Glgs103, Glgs104).  The specimens in this group differ from *N. brahma*, *N. eha*, *N. fulvescens*, *N. huang*, *N. lotipes* *N. coninga*, *N. yaoshanensis* and *N. niviventer* by the white distal tail, from *N. culturatus*, *N. andersoni* and *N. excelsior* by the small body size. They are similar to the description of *N. confucianus*, except the brown streak on back of hand and feet. | 98104, 0503131, 0812103, GLGS6893, Z201401452, Z201401453, Z201401500. |
| 29 | Animals in this lineage are medium in size (120-154 mm.). Dorsal and ventral are sharply bicolored. Dorsal and ventral are sharply bicolored. The dorsal pelage is gray brown, more black in the mid-dorsal area, slightly spinous. The fur of the back and sides is composed of three kinds of hair, (a) soft underfur is slate gray at base and brown at tip; (b) soft spines are pale white, little black at tip; (c) longer bristles black throughout. Ears medium (22-25mm.), naked, light brown. The ventral dull white throughout. Hands and feet are dull white or light browm. Tail slightly longer than head and body (117-136 % of HB), short haired, distinctly bicolored, above dark, dull white below, slightly tufted. Four pairs of mammae.  Skull similar to group 24, but more developed supraorbital ridges, more flattened braincase, zygomatic plates more forward, longer rostrum and the nasals slightly more overhanging the incisors.  Most samples/specimens from Northwest Yunnan, Southeast Qinghai and Western Sichuan, China. To include samples from Yunnan, China presented by Lu et al., 2015 (AYD498, JHB056, JHB072, JHC594, NMC670, NMC693, NMD645, NMD704, YN-GS79, YN-JC636, YN-ZD619), and from Sichuan presented by Chen et al., 2011 (CC01, CSJ07, E046, D030, D442, CSJ05, CSY05, CSJ06, E313, CSJ02, D447, CSJ03, CSY01), in which identified as *N. confucianus*.  The specimens in this group differ from *N. brahma*, *N. eha*, *N. fulvescens*, *N. huang*, *N. lotipes* *N. coninga*, *N. culturatus*, *N. andersoni* and *N. excelsior* by shot and bicolor tail, but fit with the holotype of *N. c. yajiangensis* (KIZ820284), *N. c. deqinensis* (KIZ79629) and topotypes of *N. c. yushuensis* (the same batch of holotype ) in the short and distinctly bicolored tail [[17](#_ENREF_17), [18](#_ENREF_18)]. | 0212330, Z201405613, Z201405620, 201408049, 201408066, Z20140901, Z20140902, Z20140905, |
| 30 | Animals in this lineage are variable from small to medium in size (107-162 mm.), most medium (132.06±13.93). Dorsal and ventral are sharply bicolored. The dorsal pelage is gray brown, more black in the mid-dorsal area, soft spinous or not. The fur of the back and sides is composed of two or three kinds of hair, (a) soft underfur is slate gray at base and brown at tip; (b) some have soft pale white spines (c) bristles 13-20 mm. in length, black throughout. Ears small to medium (20-24.5mm.), naked, light brown. The ventral white throughout, few with brown patch on chest. Hands and feet are white or brown streak over centre area. Tail shorter or slightly longer than head and body (87-147 % of HB), short haired and became longer hairs distally, above brown, white below and gradually trespassing on the above in distal third, most with 2-3 cm white distal tip and a white tuft, some bicolor throughout with a black tuft. Four pairs of mammae.  Skull variated from small to medium size, Supraorbital ridges low, extend backwards to the notch of parietal or to poster margin of parietals. The sides of the braincase are vertical from parietal edges to the squamosal roots. Zygomatic plates produced forward. Molars big relative to skull size. Incisive foramen well opened, their posterior ending extending backwards to or beyond the level of the first moral. Braincase slightly convex above. Bullae big and global.  Most samples/specimens from Jilin, Liaoning, Beijing, Hebei, Shanxi, Shaanxi, Gansu, Northeastern Qinghai, Hubei, Central and eastern Sichuan, Central and eastern Yunnan, China. To include samples from Qinling, Shaanxi, China presented by Jing et al., 2007 (04093, 04094), from Western Sichuan, China presented by Chen et al., 2011 (CSK02, SA564, D076, G199), and presented by Lu et al., 2015 collected from Central and Northern China, in which identified as *N. confucianus*.  The specimens in this group have various characters, but the shot tail only occur in some subspecies of *N. confucianus* [[1](#_ENREF_1), [7](#_ENREF_7), [17](#_ENREF_17), [19](#_ENREF_19), [20](#_ENREF_20)]. Some specimens fit whit the topotypes of *N. confucianus* in Muséum national d'Histoire naturelle and the photos of paratypes of *N. c. naoniuensis* (25862, 25863). | 130702, 201309003, 0509360, 0509366, 0810059, 0812147, 0905160, 0905204, 0905258, 0905390, 201408118, C204084, C204119, HNL003, Z201310045, Z201310047, Z201310049, Z201310071, Z201311074, Z201311090, Z201311096, Z201311103, Z201311124, Z201311132, Z201311133, Z201311141, Z201311149, Z201311161, Z201311164, Z201311171, Z201311181, Z201311186, Z201312227, Z201405629, |

1. Allen GM: **The Mammals of China and Mongolia**, vol. 6. New York: The American Museum of Natural History; 1940.

2. Abe H: **Variation and taxonomy of some small mammals from central Nepal**. *J Mammal Soc Jpn* 1977, **7**(2):63-73.

3. Bonhote JL: **The Mammalian Family of China.Part I.** *Proceedings of the Zoological Society of London* 1905, **2**:384-397.

4. Thomas O: **On small mammals collected in Tibet and the Mishmi Hills**. *The journal of the bombay natural history society* 1914, **23**:230-233.

5. Musser GG: **Species-limits of *Rattus brahma*, a murid rodent of northeastern India and northern Burma. American Museum novitates ; no. 2406**. In*.*: New York, N.Y. : American Museum of Natural History; 1970.

6. G. MG: **Notes on Additional Specimens of *Rattus brahma***. *American Society of Mammalogists* 1973, **23**(1):267-270.

7. Musser GG: **Notes on systematics of Indo-Malayan murid rodents, and descriptions of new genera and species from Ceylon, Sulawesi, and the Philippines**. *Bull Am Mus Nat Hist* 1981, **168**:225-334.

8. Wroughton RC: **New Rodents from Sikkim**. *The Journal of the Bombay Natural History Society* 1916, **24**(1):424-430.

9. Thomas O: **XLIV.—On mammals from the Yunnan Highlands collected by Mr. George Forrest and presented to the British Museum by Col. Stephenson R. Clarke, DSO**. *The Annals and magazine of natural history* 1922, **10**(58):391-406.

10. Thomas O: **The Duke of Bedford’ s Zoological Exploration of Eastern Asia.— XIII. On Mammals from the Provinces of Kan-su and Sze-chwan, Western China**. *Proceedings of the Zoological Society of London* 1911, **81**(1):158-180.

11. Musser GG, Chiu S: **Notes on Taxonomy of *Rattus andersoni* and *R. excelsior*, Murids Endemic to Western China**. *Journal of Mammalogy* 1979, **60**(3):581-592.

12. Li S, Yang J: **Geographic variation of the Anderson's *Niviventer* (*Niviventer andersoni*) (Thomas, 1911) (Rodentia: Muridae) of two new subspecies in China verified with cranial morphometric variables and pelage characteristics**. *Zootaxa* 2009, **2009**(2196):11.

13. Thomas O: **The Duke of Bedford's Zoological Exploration in Eastern Asia.-VI. List of Mammals from the Shantung Peninsula, N. China**. *Proceedings of the Zoological Society of London* 1908, **1908**:5-10.

14. Allen GM: **Rats (genus *Rattus*) from the Asiatic Expeditions**. *Am Mus Novit* 1926, **217**:1-16.

15. Thomas O: **On the Rat known as *Epimys jerdoni* from Upper Burma.** *The Journal of the Bombay Natural History Society* 1916, **24**(1):643-644.

16. Feng Z, Zheng C, Cai G: **A checklist of mammals of Xizang (Tibet)**. *Acta Theriol Sinica* 1984, **4**:341-358.

17. Wang S, Zheng C: **On the subspecies of the Chinese sulphur-bellied rat—*Rattus niviventer* Hodgson**. *Sinozoologia* 1981, **1**(1).

18. Deng X, Feng Q, Wang Y: **Differentiation of subspecies of Chinese white-bellied rat (*Niviventer confucianus*) in southwestern China with descriptions of two new subspecies**. *Zoological research/" Dong wu xue yan jiu" bian ji wei yuan hui bian ji* 2000, **21**(5):375-382.

19. Milne-Edwards H, Huet, Louveau, Mesnel A, Milne-Edwards A, Severeyns G, Imp. Becquet, Imprimerie de É. Martinet: **Recherches pour servir à l'histoire naturelle des mammifères : comprenant des considérations sur la classification de ces animaux**, vol. vol. 2 atlas. Paris :: G. Masson; 1868.

20. Milne-Edwards H: **Nouvelles Archives du Museum D'Histoire Naturelle De Paris**; 1871.
